# Immune defenses of the mammary gland epithelium of dairy ruminants

**DOI:** 10.3389/fimmu.2022.1031785

**Published:** 2022-10-21

**Authors:** Pascal Rainard, Florence B. Gilbert, Pierre Germon

**Affiliations:** ISP, UMR1282, INRAE, Université de Tours, Nouzilly, France

**Keywords:** mastitis, bacteria, MAMPs, PRR, epithelial cells, macrophages, innate immunity

## Abstract

The epithelium of the mammary gland (MG) fulfills three major functions: nutrition of progeny, transfer of immunity from mother to newborn, and its own defense against infection. The defense function of the epithelium requires the cooperation of mammary epithelial cells (MECs) with intraepithelial leucocytes, macrophages, DCs, and resident lymphocytes. The MG is characterized by the secretion of a large amount of a nutrient liquid in which certain bacteria can proliferate and reach a considerable bacterial load, which has conditioned how the udder reacts against bacterial invasions. This review presents how the mammary epithelium perceives bacteria, and how it responds to the main bacterial genera associated with mastitis. MECs are able to detect the presence of actively multiplying bacteria in the lumen of the gland: they express pattern recognition receptors (PRRs) that recognize microbe-associated molecular patterns (MAMPs) released by the growing bacteria. Interactions with intraepithelial leucocytes fine-tune MECs responses. Following the onset of inflammation, new interactions are established with lymphocytes and neutrophils recruited from the blood. The mammary epithelium also identifies and responds to antigens, which supposes an antigen-presenting capacity. Its responses can be manipulated with drugs, plant extracts, probiotics, and immune modifiers, in order to increase its defense capacities or reduce the damage related to inflammation. Numerous studies have established that the mammary epithelium is a genuine effector of both innate and adaptive immunity. However, knowledge gaps remain and newly available tools offer the prospect of exciting research to unravel and exploit the multiple capacities of this particular epithelium.

## 1 Introduction

The mammary gland (MG) immune system has characteristic features that are distinct from those of mucosal organs. This organ essential to the perpetuation of mammals fulfills three major functions: nutrition of the offspring by the secretion of milk, the transfer of immunity from the mother to the offspring by immunoglobulins and the delivery of immune cells, and self-defense against microbes through the homeostatic control of MG immunity. The mammary epithelium is the major actor in these three domains. The functions of milk secretion and protection of the young are beyond the scope of this review, which will focus on the self-protective role of the mammary epithelium. The MG has evolved to protect itself against pathogens without compromising offspring survival. The evolutionary process that led to the contemporary MG conserved some of its original antimicrobial properties (1), but as the nutritious function gained in importance, the MG defenses against infection were strained. The lactating MG secretes continuously a nutritious liquid that accumulates in the secretory alveoli, collecting ducts and cisterns, until discharged by suckling or milking. A major issue is the preservation of the secretory function in the face of possible colonization by pathogenic bacteria. This is a problem because milk allows certain bacteria to grow up to very high numbers (10^9^ cfu/mL) with a doubling time of less than 30 min (2). The consequence is mastitis, the inflammation of the MG, the most widespread and costly disease for dairy farming.

By definition, “the epithelium is a tightly cohesive sheet of cells that covers or lines body surfaces and forms the functional units of secretory glands” (3). Accordingly, one could infer that an epithelium consists in epithelial cells only. However, functionally, an epithelium is not limited to epithelial cells. In particular, the function of protection against microbes results from the cooperation between epithelial cells and the leucocytes that reside within or close to the epithelial lining. In this review article, full consideration will be given to this cooperation. We will consider the epithelial cell and its immediate environment, adopting the concept of an epithelial complex comprising epithelial cells and leucocytes (macrophages, dendritic cells, and lymphocytes) that reside between epithelial cells (intraepithelial leucocytes) on the luminal side of the basal membrane. Because of their unique position at the frontline of body surfaces, the cells that constitute epithelial linings are bound to be sentinels of host defense. Numerous studies have sought to elucidate how epithelia cope with their respective microenvironment, illustrating the diversity of situations and adaptive responses. When comparing the MG epithelium to the epithelia lining the gut, airways or skin, a number of key differences emerge. A major difference is that, unlike many epithelial barriers, the mammary epithelium is not directly exposed to the external environment. It is protected by the teat canal which secludes the MG lumen from its environment, except during milking or nursing. A functional teat canal delimits the intramammary environment of the MG epithelium, which is very different from the environment of the teat apex. The importance of the integrity of the teat canal is demonstrated by the impossibility to maintain a healthy lactation in a gland with a wounded teat canal, due to reiterated infections. Thus, the epithelium is the second line of defense of the MG. The MG epithelium is an immunologically active barrier that senses changes in the luminal environment, responds to intrusive agents, and interacts with resident and recruited immune cells. Another essential difference is that the lactating mammary epithelium is bathed in a nutrient medium constantly renewed in large volumes and periodically removed. Two obvious implications are that bacteria have ample fuel to proliferate in the lumen of the gland and that antimicrobial agents produced by the MECs are subjected to dilution and elimination. In this review, we will see how the mammary epithelium has evolved to meet the challenge of keeping healthy an organ that secretes large amounts of a nutrient fluid in which many pathogenic or commensal bacteria can thrive.

## 2 Setting the stage: Main features of the MG epithelium of dairy ruminants

### 2.1 Histological organisation and cell types

**Figure 1:**
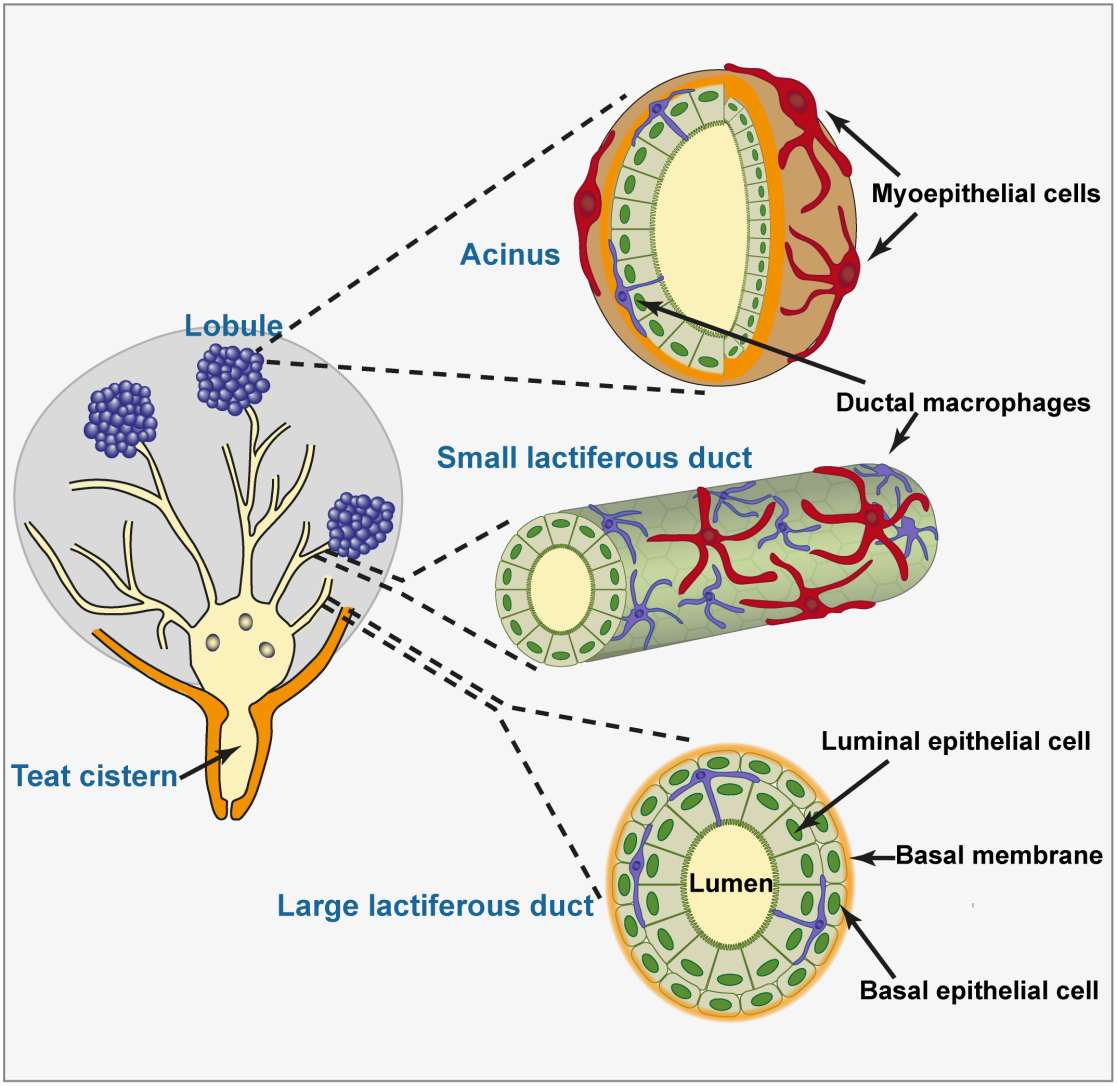
Histological organization of the mammary epithelium. The teat and gland cisterns and large lactiferous ducts are lined with a double layer of non-secretory epithelial cells resting on a basement membrane. Quite a few ductal macrophages lie between the luminal and basal layers of epithelial cells, extending dendrites that contact multiple epithelial cells and can access the lumen. Small lactiferous ducts and acini are lined with a single layer of epithelial cells. This layer is sheathed by a network of myoepithelial cells. Ductal macrophages are also present in the lobular zone, in close contact with luminal epithelial cells. The representation of ductal macrophages dendrites accessing the lumen is speculative only.

The mammary epithelium consists of one or two layers of cells sheathed by a longitudinally network of myoepithelial cells, lying on a basement membrane and an elastic connective tissue. Organized in lobules, the alveoli made up of one layer of secretory luminal epithelial cells enveloped in myoepithelial cells are connected by small ducts which open into large ducts which present a double layer of non-secretory epithelial cells (**Figure 1**). In the teat, lactiferous sinuses, and large lactiferous ducts, the epithelial lining comprises a double layer of non-secretory cells (4). These cells assume a cuboidal or columnar appearance depending on the stretching of the epithelium. There are several main types of epithelial cells in the mammary gland, the proportions of which vary depending on the physiological stages (5). Stem and progenitor cells, myoepithelial, basal and luminal cells can be distinguished by the expression of different surface molecules, keratins, and gene expression profiles. Within the epithelium, leucocytes are often observed, consisting in lymphocytes and plasma cells but not neutrophils in healthy glands (6). Macrophages together with small and large lymphocytes were commonly found between cells of both layers of the epithelium, recognizable from their structural features and the absence of desmosomes (4). Morphological appearance and two-color immunohistofluorescence have been used to identify dendritic-like cells scattered in the epithelium of alveoli and ducts, and in the sub-epithelial connective tissue (7). The characterization of the tissue-resident macrophages has been conducted in the mouse MG (8–10). Called mammary ductal macrophages, these cells form a network between the basal and luminal layers of epithelial cells and monitor the epithelium with the movements of their dendrites. They express F4/80 and CX3CR1, markers of fully differentiated macrophages. Their spindle shape and expression of CD11c and MHC class II molecules make them similar to the dendritic-like cells described in the bovine MG. The ductal macrophages are thought to play a major role in MG remodelling during post-lactational involution (8). Various subsets of ductal macrophages can be distinguished by single-cell analysis. Some seem to be poised to react to bacterial cues, and these cells are positioned to sample the gland lumen (10).

An essential property of an epithelium is its cohesiveness and tightness, conferring a role of physical barrier. The cells are joined to each other and to the myoepithelial cells by desmosomes and to the basement membrane by hemi-desmosomes. The sealing is effected by the tight junctions that are the most apical component of the junctional complex which also includes adherens junction and desmosomes (11). Tight junctions block the paracellular diffusion of ions and small molecules across the epithelium. They define the border between the apical and basolateral cell surfaces and contribute to maintain cell polarity, separating the plasma membrane into two domains of distinct protein and lipid composition (12). They are impermeable during lactation. In particular, the large ducts and sinuses are impermeable to soluble milk constituents such as lactose and even to ions (13), and plasma constituents are excluded from milk, supporting the notion of “blood-milk barrier”. The mammary epithelium is more permeable in non-lactating glands, with a rapid sealing of the mammary tight junctions at parturition.

To gain access to the lumen of the MG, bacteria have to pass the teat (or streak) canal (*ductus papillaris*). The teat canal is lined with a stratified squamous epithelium in continuity with the teat skin epithelium. At the junction of the teat canal and the teat cistern (*sinus papillaris*) the epithelium changes abruptly (squamocolumnar junction) to a bilayer epithelium. The folds of the distal rosette of the teat cistern (Furstenberg’s rosette) are considered an important place of cooperation between EC and leucocytes and could function as a primary contact site between leucocytes and bacteria. Scanning electron microscopy revealed that the MECs lining the teat cistern are densely covered with microvilli and display a characteristic hexagonal outline (6). These microvilli would hamper the passive adhesion of bacteria, and indeed bacteria adhere preferentially to cells that have lost their microvilli (14).

A feature that differentiates the mammary epithelium from epithelia frequently or permanently exposed to microorganisms is the absence of specialized epithelial cells such as Paneth cells, which release antimicrobial peptides (AMPs) or goblet cells, which secrete mucins. MECs produce membrane mucins which, unlike the mucins secreted by goblet cells in the mucous epithelium of the intestine, respiratory and reproductive tracts, are integral membrane components. The large molecular weight secreted gel-forming mucins (MUC2, MUC5, MUC6, MUC19) which constitute the mucus barriers are not produced by MECs (15). The major mucin of mouse, human, and bovine MECs is MUC1, and as an apical membrane mucin, it is also found in the fat globule membrane (16). MUC1 is a transmembrane glycoprotein with a cytoplasmic tail. It is a major constituent of the cell surface glycocalyx. Due to the richness in proline of the protein core and the rigidity conferred by heavy glycosylation, MUC1 is a rather rigid molecule, adopting a filamentous appearance that extends from the microvilli of alveolar and ductal epithelial cells (16). These filaments are prominent at the surface of milk fat globules of breast milk but less in bovine milk, in relation to the smaller size and amount of the bovine MUC1 in comparison to that of the human. The glycosylated extracellular part of MUC1 is hydrophilic, and its richness in sialic acid confers a negative charge to the cellular surface. This may help prevent the collapse of small ducts and alveoli when milk is ejected following myoepithelial contraction. It has also repellent characteristics, which may physically hamper adhesion of bacteria to MECs. On the other hand, MUC1 can bind certain bacteria such as *E. coli* or *S. aureus* (17, 18), which could favor adhesion to the epithelium lining or prevent it through the interaction with milk fat globules acting as decoy for microbial adhesins (19). The shedding of MUC1 from the cell surface can also facilitate the release of adherent bacteria (20). In the absence of a gel-like mucus layer shielding the epithelium in the MG, cell membrane-tethered mucins are likely to play an important role as a physical barrier and adhesion decoy in the defense against bacteria.

A consequence of the lack of secreted mucus covering is that MECs are directly exposed to bacteria and bacterial products. Moreover, there is no strong sub-epithelial population of plasma cells producing large amounts of IgA that are transported across the epithelial barrier to contribute to the containment of microbes at the luminal side (21). This characteristic distinguishes the mammary epithelium from the upper airways or digestive tract epithelia that accommodate metabolically active microbiota.

### 2.2 Strengths and weaknesses of experimental protocols used to investigate the epithelium response to bacteria

The MG comprises a variety of cell types that may contribute to the immune competence of the udder (22). As most cells of the epithelium lining are epithelial cells, it is of major interest to characterize the responses of these cells to pathogens. *In vitro* bacterial-epithelial co-cultures have been used extensively to elucidate the mechanisms by which bacteria adhere, invade, and signal to the host, and to examine epithelial cell responses. The biological relevance of these studies relies on appropriate gene expression and cellular functioning of both the microbial and host cells. It is therefore critical that representative host cells are exposed to bacteria or bacterial products under conditions that mimic *in vivo* situations as closely as possible. The complexity of the mammary tissue is virtually impossible to reproduce *in vitro*, but more or less sophisticated experimental models have been used to obtain valuable information. Investigators using these models need to be aware of each model limitations to interpret correctly their data, as a number of essential issues need to be taken into account (**Table 1**), as pointed out early on (23).

**Table 1:** Critical aspects in the *in vitro* analysis of MEC response to bacteria.

**Primary cultures of MECs**
Culture conditions: solid substrate, extracellular matrix, growth factors, complex medium, presence of serum, of milk Number of passages, level of differentiation, dedifferentiation, loss of pristine characteristics Polarization, presence of tight junctions, culture on porous membrane Variability of batches and individual sources Alveolar versus ductal EC
**Mammary cell lines**
Same issues as with primary cells, except variability Genome modifications, degree of differentiation Representativeness: loss of properties, acquisition of others
**Bacteria**
Live bacteria: shedding of bacterial products and metabolites but depletion of nutrients, oxygen, and medium acidification, imposes low multiplicity of infection and short exposure duration Killed bacteria: no metabolism, inert bodies, but exposure duration can be long without cell damage Choice of strains, culture condition determining phenotype
**Bacterial products**
Complex mixtures: culture supernatant, bacterial extracts, variety of conditions Purified MAMPs: degree of purity/contamination by other MAMPs
**Cell co-cultures and tissue explants**
Takes into account the interrelationship between different cell types that comprise the MG Difficulty: relevance of the leucocytes used in co-cultures with MECs

Primary cultures of MECs from dairy ruminants are relatively easy to establish. After isolation, MECs can be cultivated, replicated under controlled conditions, cryopreserved, thawed and re-cultured for several passages. However, maintaining cultured cells in terminally differentiated state is difficult. The substrate on which the cells are grown is essential, as shown by the effect of extracellular matrix on the production of milk components by cultured cells (24, 25). Unfortunately, the effect of growth substrata on the immune capacity of MECs remains largely undefined. A combination of factors such as insulin, epithelial growth factor, prolactin, cortisol and fetal calf serum (FCS) has been shown to be essential for growth and differentiation of MECs (23). The age of the culture, the stage of confluence, the maturation and aging of the cells are likely to influence the results of experiments. The number of passages of primary cells interferes with their reactivity, as shown by the possible loss of lingual antimicrobial peptide (LAP) expression after a few passages, underlining the need to use short-time subcultures to analyze MEC defense genes (26). Large batches of cryopreserved cells allow investigators to overcome this impediment. Another limitation is the variation between batches of cells and the individual cell donor in response to a given stimulus. This applies also to cell lines, which are derived from one animal, thus providing information limited to one genotype. Cell lines either intentionally [e.g. MAC-T cells [27)] or spontaneously [e.g. PS cells (28)] transformed, are expedient because they can be used on a long period of time and can be exchanged between laboratories, thus facilitating comparisons of experimental data. However, their transformation may alter their behaviour. For example, it has been reported that the bovine cell lines MAC-T cells are deficient in LAP and inducible nitric oxide synthase (iNOS) expression (29) and the BME-UV1 cell line may lack some functional lipopolysaccharide (LPS)-responsive elements (30). This is why ideally studies based on one or several cell lines should also involve primary cells derived from several animals.

The composition of the medium in which MECs are exposed to the stimuli, i.e. the stimulation medium, influences the behavior of the cells. Although glucocorticoids are essential hormones for mammary secretory activity (31), they are likely to interfere with the immune response of MECs. One way to reduce their influence is to reduce their concentration in the stimulation medium (32, 33). The presence of fetal calf serum during stimulation has been shown to augment the response of MECs to *E. coli* but to reduce the response to *S. aureus*, leading to discrepant results (32, 34). MECs do not bathe in blood plasma, but they have access to exuded plasma components at their basal side, and bathe in milk at their apical side. Importantly, interactions of MECs with bacteria and bacterial components or metabolites take place at the apical side in the absence of serum and in the presence of MG secretions, milk or “dry secretion”. It can be argued that deprivation of serum during stimulation stresses the cells, but that stimulation in the presence of serum is artificial. Raw milk is the best approximation of the *in vivo* environment. Skim milk is not perfect, as shown by the inhibition of adhesion of *E. coli* to MECs by whole milk but not by skim milk, supposedly because *E. coli* would interact with milk fat globules (35). Skim milk reconstituted from powder milk has been shown to quench the response of MECs to *E. coli* and *S. aureus* (34), whereas whole fresh milk augmented the response of MECs to *E. coli* similarly to the addition of recombinant soluble CD14 (sCD14) to the stimulation medium (36). The limitations of each medium should be kept in mind when interpreting the data.

The mode of presentation of bacteria is of prime importance. In principle, the use of live bacteria is the best approximate to MG infection. The major limitation is that bacteria have to be removed or their growth halted to prevent overgrowth, nutrient and oxygen depletion, and acidification of culture medium. An expedient solution is to wash the cell culture after 3 h of co-culture and replace the culture media with fresh medium containing an antimicrobial (32, 37). However, this short time impedes the production and shedding of bacterial agonists of the innate immune system resulting from bacterial growth, and precludes the study of reaction of MECs to prolonged co-culture. The use of killed bacteria circumvents the problem posed by live microorganisms, but ignores the possible crucial effects of bacterial virulence factors, possibly induced by the MEC/pathogen interactions. Besides, the effects of adherence and invasion on host cells are not fully taken into account if necessitating an active part from the pathogens. In every case, the relationships with experimental infections of the MG are limited to the initial phase, mainly the triggering phase of the innate immune response. This addresses imperfectly the contribution of the mammary epithelium to the resolution or chronic phases of infection.

An alternative to live or killed bacteria is the use of bacterial culture supernatants, exosomes, crude or purified MAMPs. This approach may partially recapitulate the stimulus exerted by bacteria proliferating in the MG lumen, but it is not free from criticism. These products may not be representative of the proteome and metabolites produced during infection. In addition, this approach is not adapted to all pathogens: during growth in culture media, staphylococci release many components (proteins, lipoproteins, polysaccharides, and lipopolysaccharides) while streptococci release much fewer compounds, as well as *E coli* which sheds very few soluble components but release exosomes called outer membrane vesicles (OMVs) (38). *S. aureus* also sheds extracellular vesicles that contain MAMPs that stimulate MECs (39). However, the use of purified or synthetic compounds makes it possible to focus on specific PRRs and to establish the repertoire of MAMPs sensed by the cells under study (40). This is also convenient because dose-responses can be established. It should be kept in mind however that MAMPs may not be shed or accessible on intact bacteria as for example the lipoteichoic acid (LTA) of *S. uberis* (41). The importance of the physical characteristics of the stimuli is also illustrated by a study showing that genes associated with oxidative stress were more upregulated after live bacteria stimulation, whereas immune response related genes were more highly expressed after supernatant stimulation in the early phase of exposure (42).

On solid substrata, MEC monolayers are poorly polarized. Expression of PRRs may not be equal on basal and luminal sides of the cells under physiological conditions. A more representative system is when MECs are cultured on a porous membrane with a collagen cushion: the cells develop tight junctions (high trans-epithelial resistance) and are likely polarized (43). Fibroblasts can also be added in the collagen cushion. Nevertheless, this model does not reproduce the *in vivo* situation. An even better model is the tissue explant, which preserves the cellular composition and the architecture of the MG, allowing investigations in a native environment. Although explant cultures have been used to study mammary tissue growth requirements, they have seldom been used to investigate interactions with bacteria (23, 44).

Whatever the usefulness of *in vitro* models, *in vivo* experiments with luminal infusion of agonists (microbe-associated molecular patterns, MAMPs) of the innate immune receptors (pattern recognition receptors, PRRs) are a convenient and relevant method to test the reactivity of the epithelium lining the MG lumen. The MG comprises a variety of cell types that may all contribute to the immune competence of the udder. The decisive advantage over *in vitro* experiments is that the cells that make up the epithelium function in their natural environment and can cooperate with each other. Precision-cut udder slices can be used to investigate the reaction of the mammary tissue to MAMPs or live bacteria (45, 46). A limitation is that the slices expose parts of the tissue that are not directly exposed to bacteria during natural infection. An intermediary ex vivo model based on isolated perfused bovine udders has been used to study the early stage of inflammation of mammary tissue (47, 48). Experimental infection of the MG is the most relevant model, but due to the complexity of interactions with recruited leucocytes, it is difficult to unravel the contribution of the epithelium. The comparison of *in vitro* and *in vivo* models is however indispensable to define the relative role of MECs, the epithelium, and the cells recruited by inflammation.

## 3 Sensing bacteria

### 3.1 MAMPs detection by the mammary epithelium

The recognition of the bacterial threat is a prerequisite to the initiation of immune responses and the mobilization of defences. Innate immune recognition of bacteria involves a limited number of PRRs that recognize conserved microbial molecules, referred to as MAMPs (49). The most widely studied PRRs and primary sensors of bacteria are the Toll-like receptors (TLRs), transmembrane proteins that recognize microbial compounds with defined structural features (**Table 2**). Other important sensors are in the cytosol, like the nucleotide-binding oligomerisation domain (NOD)-like receptors (NLRs) NOD1 and NOD2 (68, 69). NOD1 and NOD2 are cytoplasmic proteins that detect bacterial peptidoglycan elementary fragments. NOD1 reacts to iE-DAP, a dipeptide present in peptidoglycan primarily found in Gram-negative bacteria, whereas NOD2 reacts with MDP that is present in all bacterial peptidoglycans. The question arises of how intact extracellular bacteria can be detected by cytosolic sensors of small peptidoglycan fragments. During bacterial cell division, a sizeable amount of peptidoglycan is released as small fragments (70). Bioactive fragments of peptidoglycan are released in the environment during bacterial growth (71). The natural ligand of NOD1 is released in the culture supernatant of *E. coli* (72). Epithelial cells may use oligopeptide transporters, such as the pH-sensing regulatory factor of peptide transporter 1 (PEPT1) and solute carrier family 15 member 4 (SLC15A4) to bring peptidoglycan fragments into the cytosol (70, 73). Of note, Tri-DAP, which is a hydrophilic molecule, does not trigger inflammation in the MG, whereas C12-iE-DAP, which is rendered membrane-permeable by its lipophilic moiety, does (40). Alternatively, peptidoglycan from non-invasive *E. coli* is delivered to cytosolic NODs through OMVs, which are internalized *via* endocytosis (74).

**Table 2:** Reactivity of MECs and the MG to bacterial agonists of the innate immune system.

PRR Accessory molecules	MAMP recognized	Expression by MECs or presence in milk	*In vitro* reactivity	MG (*in vivo*) reactivity
TLR2/TLR1 heterodimer	Triacylated lipopeptides and lipoproteins Model MAMP: Pam2CSK4	mRNA (50)	Yes (40)	
TLR2/TLR6 heterodimer CD36	Diacylated lipopeptides and lipoproteins Model MAMP: Pam3CSK4	Yes (33) (51)	Yes (40)	
TLR2/?	Lipoteichoic acid (LTA)	Yes (52)	Yes + (33, 53)	Yes ++ (54)
TLR3	Double-stranded RNA Polyinosinic –polycytidylic acid (polyI:C)	?	?	?
TLR4 mCD14 sCD14 LBP CD36	Lipid A part of Lipopolysaccharide (LPS)	Yes (55) No (28, 56) Yes (57, 58) Yes (57, 59) Yes (60)	Yes ++ (with milk or serum)	Yes +++
TLR5	Flagellin	Low if any (40)	No	No
TLR7 and TLR8	Fragments of single stranded RNA Imidazoquinolines, “vita-PAMPs”	?	?	?
TLR9	Single stranded DNA with unmethylated CpG	mRNA (61)	No (62)	
NOD1 (CARD4)	γ -D-glutamyl-meso-diaminopimelic acid (iE-DAP)	mRNA (40)	Yes (40)	Yes (40)
NOD2 (CARD15)	Muramyl dipeptide (MDP)	mRNA (33, 63)	No (33)	Yes +++ (33)
C-type lectin receptors Dectin-1	β-glucan	Yes (rat MEC) (64)	Yes (rat MEC) (65)	Yes (66)
Inflammasome NLRP3	Uric acid, extracellular ATP, invasive bacteria	Yes (67)	Yes (67)	?

?: unknown.

+/++/+++ : increasing reactivity level.

MAMPs associated with common mastitis-associated bacteria are components of the bacterial cell envelope and cell wall, such as the outer membrane LPS of Gram-negative bacteria, the LTA of Gram-positive bacteria, and peptidoglycan fragments. The reactivity of cells or tissues to MAMPs can be tested conveniently with model agonists of PRRs. It has long been known that the MG of ruminants is very sensitive to *E. coli* LPS (75, 76). The amount of LPS that triggers the influx of neutrophils into the lumen of a lactating MG is as low as 0.2 µg, despite the dilution in at least 40 mL residual milk (77). Intraluminal instillation of staphylococcal LTA also triggers inflammation, although much higher concentrations (one or two orders of magnitude) than those of LPS are necessary (54). These experiments have shown that exposure of the MG epithelium to bacterial components triggers an inflammatory response. Being the most numerous cells directly exposed to bacteria, MECs are likely actors of this reaction. This is why the contribution of MECs to sensing of MAMPs by the MG has been extensively studied *in vitro*.

### 3.2 The sensing capability of MECs

Experiments with cultured MECs showed that these cells possess the molecular machinery necessary to sense and respond to the most common mastitis-associated bacteria. Incubation of primary bovine MECs (pbMECs) with *E. coli* or *S. aureus* induces the overexpression of chemokines and inflammatory cytokines mRNA transcripts (32). However, pbMECs are less apt at sensing *S. uberis* (41). Bacterial MAMPs such as LPS and LTA elicit pbMEC responses (53). The mRNA expression for TLR2, TLR4 and TLR9 was found in bovine mammary tissue of healthy glands (61). At MEC level, mRNA expression for TLR1, TLR2, TLR4, TLR6, NOD1, and NOD2, but not TLR5 has been documented (40). This finding correlates with the reactivity of the mammary gland to instillation with LPS, LTA, Pam3CSK4 (synthetic lipopeptide agonist of TLR2/6), Pam2CSK4 (agonist of TLR2/6), C12-iE-DAP (agonist of NOD1), but not with flagellin (agonist of TLR5) (40, 54). Of note, bMECs react more strongly to Pam2CSK4 than to Pam3CSK4, consistent with the higher expression of TLR6 than TLR1 mRNA. This suggests that the MG could react more vigorously to diacylated than to triacylated lipoproteins. The reactivity of the MG and bMECs to MDP (agonist of NOD2) has also been shown (33).

The case of flagellin deserves to be examined, because as this MAMP has a prominent role in the recognition of mucosal pathogens (78), the lack of reactivity of the MG was unexpected. The flagellin used was from *Salmonella enterica* serovar Typhimurium (40), known to activate most mammalian TLR5 receptors, and the motif recognized is shared by *Salmonella* and *E. coli* (79). Although slightly different from its human counterpart, the bovine TLR5 is fully functional (80). As the TLR5 gene is weakly transcribed by bMECs (40), the failure of response to flagellin likely results from very low expression of TLR5 by bMECs and its inaccessibility from the lumen of the gland. The lack of reaction of the MG to intraluminal exposure also suggests that intraepithelial leucocytes do not sense this MAMP.

A key issue is the ability of MECs to react to bacterial components released into the gland lumen, i.e. from the apical side of the cell. Epithelial cells are polarized, with a basal and apical sides that are not identical in terms of cell membrane composition. We have little information on the polarization of TLR expression on MECs. The reactivity of the MG to luminal instillation of MAMPs is an indirect evidence that PRRs are accessible to MAMPs present in the gland lumen. However, expression of TLRs at the apical surface of MECs seems to be rather low in healthy glands, as immunohistochemistry analysis of mammary tissue did not reveal expression of TLR2 or TLR4, contrary to strong expression early after infection or LPS challenge (56, 81). However, TLR2 and TLR4 have been found by confocal microscopy at the apical membrane of bovine alveolar MECs and TLR4 in apical position of bMECs grown on a porous membrane coated with collagen (82, 83). Upon exposure to *E. coli* LPS, the apical expression of TLR4, but not TLR2, was transiently augmented by mobilization from the cytoplasm compartment (83). The levels of expression of TLR1, TLR2, TLR3, TLR6, and TLR9 by pbMECs increase after infection by *Mycoplasma bovis* (84).

At the cell membrane, PRRs that interact with MAMPs are aided in their function by accessory molecules (**Table 2**). Bacterial cell wall amphiphilic MAMPs, such as the LPS of Gram-negative bacteria and the LTA of Gram-positive bacteria, are recognized *via* their lipid anchor to TLR4 and TLR2, respectively (52), although the identity of PRRs involved in LTA recognition is under debate (52). Accessory molecules and co-receptors concentrate microbial products on the cell surface (85). One of the co-receptors is the transmembrane lipid scavenger protein CD36 which binds diacylglycerol ligands and transfers them to the accessory molecule CD14, which ultimately loads the ligands onto TLR2/TLR6 heterodimers (51). CD36 is expressed by MECs and cooperates with TLR4 to react to *E. coli* LPS (60). Besides LPS and LTA, lipoproteins are very active MAMPs. Lipoproteins from Gram-negative bacteria typically have three lipid chains, and most of them are associated with the outer membrane. Lipoproteins from Gram positive bacteria are generally only diacylated and associated with the plasma membrane (52). Lipoproteins activate TLR2, usually with the contribution of co-receptors, such as the co-receptor membrane CD14 (mCD14) or soluble CD14 (sCD14), and the scavenger receptor CD36 for triacylated lipoproteins, with the notable exception of the model lipopeptides Pam_3_CSK4 and Pam_2_CSK4 (86). Most epithelial cells are CD14 negative and poorly responsive to purified LPS (87). In the MG, CD14 was not detected on MECs by immunohistochemistry (56) and on pbMECs in culture (28). Soluble CD14, which can be provided by serum, enables these cells to respond to LPS. In human milk, sCD14 is found in concentration 20-fold higher than in serum, and it is released by human MECs (57). In bovine milk, sCD14 is present at moderate concentrations, which rise in inflamed MGs (28, 88). Bovine MECs need sCD14 to react to *E. coli* LPS (28, 58).

The myeloid differentiation protein 2 (MD2), which is associated with the extracellular domain of TLR4, enhances the response of TLR4 to LPS (89). Its expression at the protein level by bMECs is not documented, but MD2 expression (mRNA and protein) in MG tissue (Holstein cows) has been reported, with a decreased expression during mastitis (90). In addition, CD14 is necessary to smooth LPS (S-LPS) recognition and activation of TRAM-TRIF-dependent signalling (91). In the MG, TLR4 is expressed at the apical side of MECs (83), and its moderate expression may be compensated by the presence of the scavenger receptor CD36 in the membrane of MECs, and of sCD14 in milk. TLR4 activity is helped by the LPS binding protein (LBP), a serum acute phase protein that enhances the transfer of LPS to CD14 (92). The concentration of LBP is very low in human milk (57), and the concentration of LBP in cow’s milk is about one-sixth of its concentration in blood (59). The addition of human LBP to bovine milk did not improve the already high response of bMECs to LPS in the presence of sCD14 (28).

**Figure 2:**
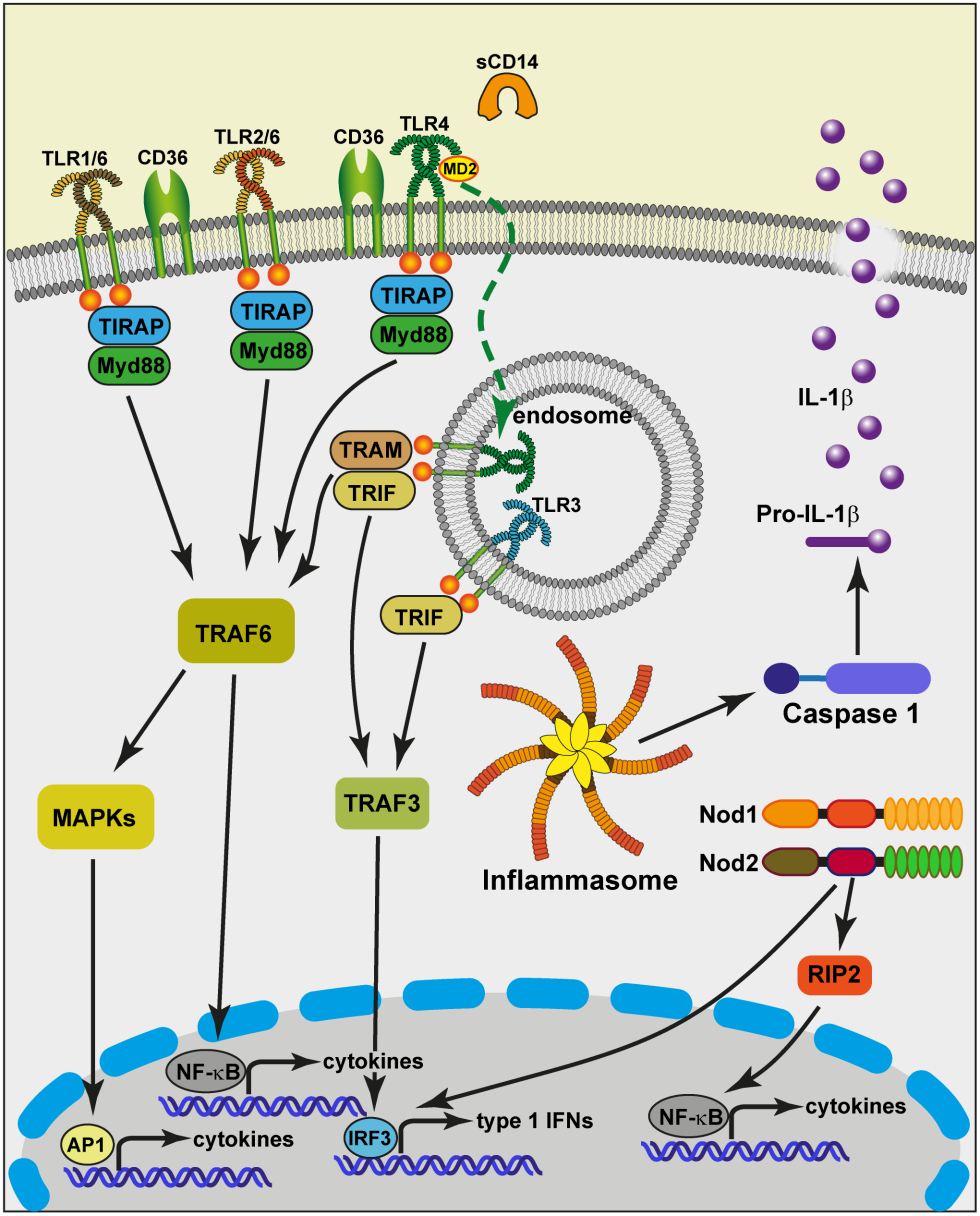
Schematic view of the potential capacity of MECs to sense and react to bacteria that invade the MG lumen. At the apical side of the cell, the plasma membrane exposes Toll-like receptors (TLR1, TLR2, TLR6, TLR4) that pair to interact with bacterial lipoproteins and LPS. The TLRs receive help from accessory molecules such as CD36 and MD2. TLR5 has not been documented in relation to MECs. MECs are devoid of membrane CD14, but milk provides soluble CD14 (sCD14). This allows TLR4 to be internalized upon ligation to smooth LPS in endosomes from where the TRAM-TRIF adaptors can be recruited to activate TRAF3. TLR3 from endosomes can also activate TRAF3. The TRIF-dependent signaling pathway induces the translocation of IRF3, resulting in the induction of type 1 IFNs and IFN-inducible genes. Contrary to TLR3, the other TLRs depend on the adaptor molecule Myd88. This triggers a cascade of activating steps involving TRAF6 and allows NF-κB to translocate into the nucleus and activate the transcription of cytokine genes. Another activation pathway mobilizes the MAP kinase cascade and leads to the activation of the transcription factor AP-1, critical in the activation of cytokine genes. MECs can respond to degradation products of bacterial cell wall peptidoglycan *via* the cytosolic sensors NOD1 and NOD2. The oligomerization of these sensors induces the recruitment of the adaptor protein RIP2, followed by the activation of the NF-κB pathway (93). A number of stimuli induce the formation of molecular platforms called inflammasomes. The NLRP3 inflammasome recruits Caspase 1 that can cleave pro-IL-1β molecules and contribute to the secretion of the mature pro-inflammatory cytokine IL-1β. TRIF, Toll/IL-1 receptor (TIR) domain-containing adaptor protein inducing IFNβ; TRAM, TRIF-related adaptor molecule; IRF3, IFN-regulatory factor 3; Myd88, myeloid differentiation primary-response protein 88; NOD, nucleotide-binding oligomerisation domain; RIP2, Receptor-interacting-serine/threonine-protein kinase 2.

Overall, it seems that the constitutive expression of PRRs and accessory molecules by MECs is limited (**Figure 2**): there is a low level of expression of TLR2, TLR4, no expression of TLR5 and mCD14. The expression of some TLRs (TLR2, TRL4), and accessory molecules (sCD14, LBP) is inducible and greatly enhanced by exposure to bacteria (55, 56, 61, 94, 95). This overexpression may be transient, resulting in a phase of increased reactivity of the MG to bacteria, tempered by the activation of inflammation regulation mechanisms. It could be that intraepithelial leucocytes compensate for the suboptimal constitutive expression of PRRs by MECs.

### 3.3 Cooperation of MECs with macrophages

Although it is undeniable that MECs participate in the innate immune response to infection, they are not the only cells that contribute. The various responses of MECs to different pathogens, such as the abrupt inflammatory response caused by *E. coli* and the sluggish reaction induced by *S. uberis* paralleling the *in vitro* response of MECs to these pathogens, relate to the pathogen-specific physiopathology of mastitis (29). However, a number of discrepancies between the responses of MECs *in vitro* and the *in vivo* response of the MG to the same stimuli have been noted (**Table 2**). Both *S. uberis* and *S. aureus* MG infections often provoke intense inflammatory responses in the few days following initiation of infection (96), despite the inability of bMECs to sense *S. uberis* (41). The luminal instillation of MDP elicits an intense influx of leucocytes with the early secretion of neutrophil-targeting chemokines and pro-inflammatory cytokines TNF-α and IL-1β whereas *in vitro* exposure of pbMECs to MDP does not (33). There is a discrepancy between the expression of immunity-related genes in the mammary tissue of *S. uberis*-infected quarters and in MECs exposed to the same bacteria (97). Bovine MECs and monocyte-derived macrophages also do not react the same way to *S. uberis* exposure (29). The very low amount of TNF-α secreted by bovine MECs exposed to live *E. coli* (32) and the absence of secretion of IL-1β despite upregulation of the gene transcripts suggests that the triggering of inflammation involves other cell types. Resident macrophages are likely candidates, and the role of these cells has been investigated with a mouse mastitis model. Depletion of alveolar and epithelial macrophages with liposome-encapsulated clodronate, an agent that inactivates phagocytes, and the use of a set of knock out mice for TLR4, TNF-α or IL-1β has been used to establish that macrophages were necessary to elicit neutrophil recruitment into the MG lumen in response to LPS infusion, in relation with the TNF-α produced by macrophages in response to LPS/TLR4 signalling (98). The PMN recruitment occurred when macrophages were inactivated during infection with live *E. coli* but the bacteria were able to invade MECs and develop intracellular microcolonies (99). Due to the small number of alveolar macrophages, the authors proposed that they operated, possibly through TNF-α secretion, in a paracrine and autocrine manner on MECs (98). We do not know if these findings apply to the cow. In the healthy MG, alveolar macrophages are few, and they are bound to be shed with the milk at each milking. Moreover, milk macrophages are not very responsive to innate or adaptive immune stimuli [discussed in (100)]. Better candidates of immune reactivity are the ductal macrophages that populate the bovine (7) and murine (8, 10) mammary epithelium. These cells present a high expression of CD14, CD11c, and major histocompatibility class II (MHC-II) markers. Besides their likely capacity of antigen-presenting cells, they are also likely to play a role in innate immunity. Located just beneath the layer of luminal MECs, these cells display numerous and elongated dendrites that make them particularly apt at sampling the MG lumen. Their close contact with MECs suggests that they exchange information with them. The contribution of ductal macrophages to the innate and adaptive defence of the MG, and in particular to the sensing of pathogen, is of major interest. The crosstalk between MECs and macrophages is likely to condition the reactivity of both cell types to pathogens (101). In the mouse, the production of colony stimulating factor 1 (CSF-1) and TGF-β1 by MECs has been shown to modulate the activities of mammary macrophages (reviewed in [102)]. Although much remains to be discovered about the interplay of MECs and ductal macrophages, it can be argued that these interactions play a crucial part in the initial and protracted response of the MG to infection. This line of research, which has been neglected so far, deserves more attention.

### 3.4 How the mammary epithelium “sees” bacteria

Bacterial invaders comprise an array of MAMPs, so that several epithelial sensors are likely to be involved simultaneously. Consequently, different signaling pathways are activated, which may lead to an additive or more than additive (synergistic) response. This is likely to occur in the MG. When infused into the lumen of the MG, MDP and staphylococcal LTA exert a synergistic effect to induce neutrophilic inflammation (33). The two MAMPs in combination induce higher secretion of chemokines by bpMECs than when used alone. Staphylococcal LTA and peptidoglycan, but not LTA or peptidoglycan alone, activated MAC-T cells (103). Synergistic effect of MAMPs on bMECs has been shown to induce a strong transcriptomic response including inflammation-associated genes and a decrease in casein gene expression (104). Moreover, bacteria are not inert “bags of MAMPs” (105). MAMPs are usually embedded in the bacterial cell wall or outer membrane and shielded by polysaccharide structures, so that intact bacterial bodies are hardly perceived by cell surface PRRs. As a result, bacterial bodies are poor inducers of innate immune responses, unless they are ingested and processed in phagolysosomes. Bacterial surface MAMPs need to be extracted to be available for PRRs. This is the role of scavenger molecules that can transmit the extracted compounds to the co-receptors that, in turn, ferry them to cell surface PRRs. This is particularly important for bacteria that secrete little during growth like *S. uberis* or in insoluble form like *E. coli*. Extraction of LPS from the outer membrane to make the lipid A moiety accessible is a prerequisite to interaction with TLR4. Molecules such as LBP, lipoproteins or serum albumin (106) are supplied by serum and their presence or absence in the tests is of key importance although often unheeded by experimenters. This may be less important for bacteria like *S. aureus* which tend to profusely secrete many soluble proteins. Moreover, both Gram-positive and Gram-negative bacteria shed membrane vesicles, nanoparticles composed of lipid membranes that encompass many bacterial components such as MAMPS and virulence factors (107). For example, *S. aureus* extracellular vesicles elicit inflammation in the murine MG (39), and can stimulate pbMEC *in vitro* (108). The precise mechanisms of PRR activation upon exposure to bacterial bodies or exosomes are still poorly understood. Regardless, bacteria that signal themselves with released products must be metabolically active to be detected.

In general, the innate immune system tends to respond strongly to bacteria that multiply actively in the infected organ or tissue (109). As the basic requirement for bacteria to endanger the MG integrity is the capacity to proliferate in MG secretions, we can hypothesize that what is detected and needs a prompt response is metabolically active bacteria: bacterial growth can be rapidly overwhelming in lactating MGs and poses a real threat to its secretory function. The concept of viability-associated (vita)PAMPs, molecules produced by viable but not dead microbes, pathogens or not, such as bacterial messenger RNA (bRNA, that lacks a poly(A) tail), quorum sensing molecules, peptidoglycan fragments release during growth or lysis, or prokaryotic metabolites (110, 111), meets this hypothesis. In addition, activation of PRRs by distinct ligands and within distinct cellular compartments determines the degree of inflammation: cell surface, vesicular, and cytosolic PRRs are involved in increasingly threatening infections, and their involvement escalates the inflammatory response (112). For example, ingestion of bacteria and release of bRNA into the cytosol is usually required for activation by vita-PAMPS. A result is the activation of the inflammasome NLRP3 with the upstream involvement of TRIF and IRF3 activation (111). More generally, the MAMPs that activate the inflammasome do so upon delivery to the cytosol and not from the cell surface (105). This is why the issue of ingestion of bacteria by MECs is crucial, as a first step of cytosol contamination.

## 4 Reacting to bacteria

### 4.1 Response of MECs to bacteria or bacterial components

**Figure 3:**
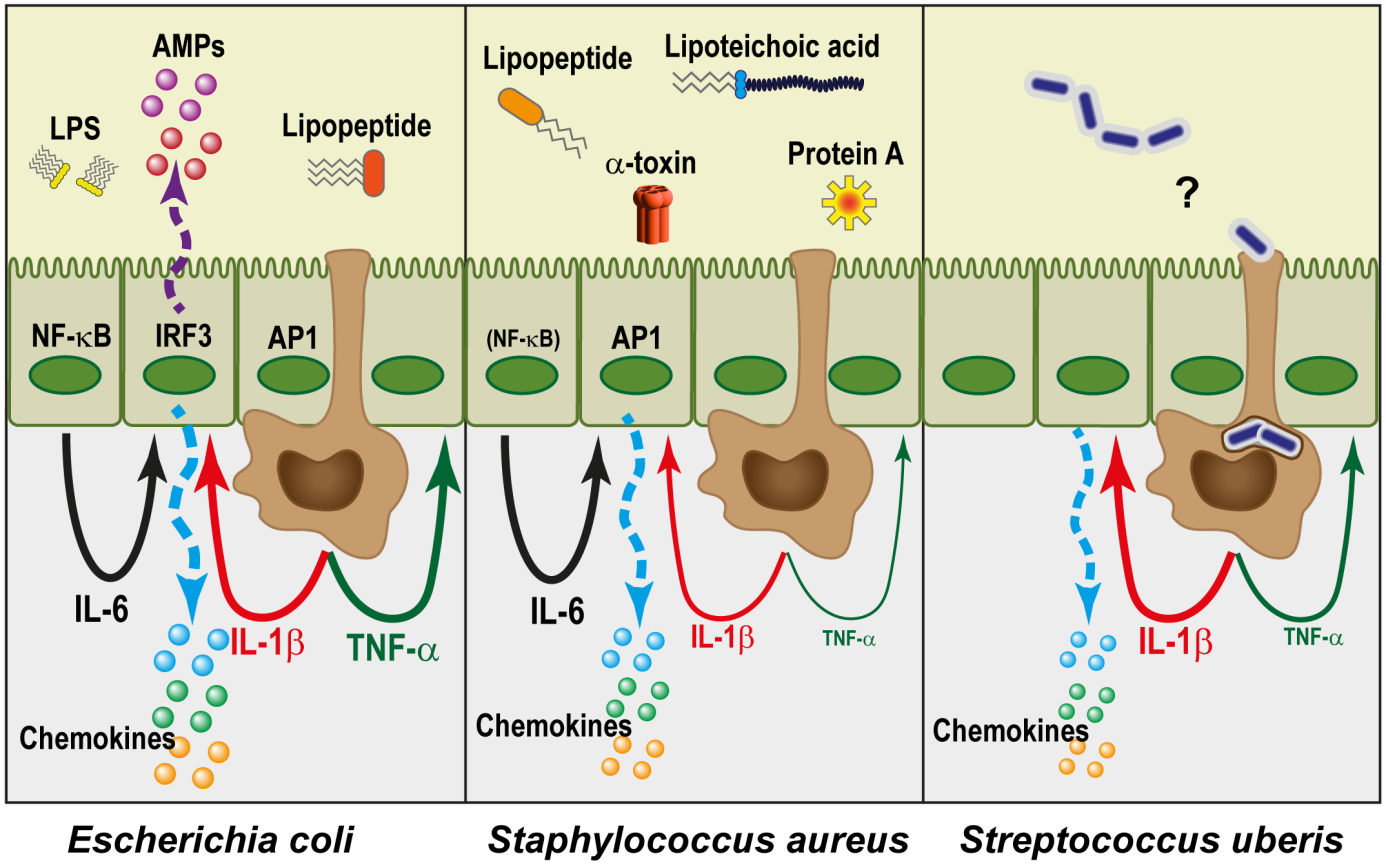
The mammary epithelium reacts differently to different pathogens. Gram-negative bacteria such as *E. coli* are essentially perceived through the lipid A moiety of LPS (endotoxin), and in combination with lipopeptides they activate the transcription factors NF-κB, AP-1, and TRF3. This leads to the expression of a large number of genes. In particular, there is a high production of chemokines and antimicrobial peptides (AMPs) by MECs, and the pro-inflammatory cytokines TNF-α and IL-1β by macrophages (brown cells). Together with IL-6 which can be produced by MECs, this results in an amplification of the self-defence of MECs and recruits a high number of circulating leucocytes, while strongly reducing their milk secretory activity. Gram-positive bacteria, which lack endotoxin, induce a comparatively lesser reaction, especially from MECs. *Staphylococcus aureus* is perceived through lipopeptides, lipoteichoic acid, protein A, α-toxin and other components and metabolites. Despite the variety of agonists, the production of IL-1β, and especially of TNF-α, is much lower than in the case of *E. coli*, presumably in relation to a much weaker activation of NF-κB. Nevertheless, the AP-1 pathway works, and IL-6 is produced. As a result, recruitment of leucocytes by chemokines takes place, but activation of self-defence mechanisms is limited. The case of *Streptococcus uberis* remains somewhat mysterious since the MECs do not seem to detect them. The late but sometimes intense reaction that these pathogens induce in the udder could result from their phagocytosis by ductal macrophages, which remains to be established.

Once alerted to bacterial presence, MECs readjust their secretory activity and perform innate immune effector functions (**Table 3**). The cellular responses are driven by the combination of the individual sensors that are activated. The response of MECs varies according to the stimulating bacteria, reflecting the activation of different PRRs (**Figure 3**). In a second step, the response is modulated by the cytokines that are produced by MECs themselves in an autocrine or paracrine manner, and by resident and recruited leucocytes. In this section, we will consider the response of MECs, independently of leucocytes.

**Table 3:** Main responses of MECs to bacteria and MAMPs.

Types of responses	References
**Self defence**	
iNOS (production of NO) and ROS (lactoperoxidase) Anti-microbial peptides (β-defensins and cathelicidins): BNBD5, LAP, TAP Lactoferrin, Complement components (C3, factor B, C4BPα) Acute phase proteins (Haptoglobin, SAA3, PTX3) S100 calcium binding proteins (S100A8, S100A9, S100A12) Phagocytosis and autophagy	(113–115) (116–120) (121–124) (125–131) (128, 131–133) (134)
**Chemoattraction of blood leucocytes**	
ELR-CXC chemokines (attract neutrophils): CXCL1, CXCL2, CXCL3, CXCL5, CXCL8 CCL chemokines (attract mononuclear leucocytes): CCL2, CCL5, CCL20	(135–138) (136, 137, 139, 140)
**Modulation of inflammation**	
Production of pro-inflammatory cytokines (IL-6, IL-1α, IL-1β, TNF-α) Production of lipid mediators of inflammation (oxylipids) Regulation of MECs & leukocyte response (TGF-β)	(32)} (136, 137) (141) (103, 142, 143)
**Modulation of barrier leakiness**	
Increased tight junction permeability (in response to LPS, IL-1β, TNF-α) MEC exfoliation, apoptosis, sloughing Tight junction recovery	(144–146) (147, 148) (149)
**Reduced milk component synthesis and secretion**	
Reduced production of caseins by TNF-α, IL-1β) Reduction in milk fat synthesis Effect of NF-κB activation	(150) (104) (151)

BNBD5, bovine beta-defensin 5; C4BPa, complement C4 binding-protein 4; iNOS, inducible nitric oxide synthase; PTX3, pentraxin 3; ROS, reactive oxygen species; SAA3, serum amyloid A3.

#### 4.1.1 Reacting to *E. coli*

Many studies have examined the response of MECs to live or killed *E. coli* or to *E. coli* LPS. These responses can be considered benchmarks against which responses to other mammary pathogens can be compared. Although studies differed in some findings, likely resulting from different stimulation protocols, characteristic responses of MECs to *E. coli* or LPS can be identified. Early studies showed that MECs react to *E. coli* by overexpressing genes for chemokines and cytokines. During *E. coli* mastitis, MECs lining alveoli and ducts were shown by *in situ* hybridization to produce CXCL8 mRNA (152), converging with the *in vitro* production of CXCL8 by MECs in response to *E. coli* LPS (135) to establish that MECs are a major source of CXCL8 during *E. coli* mastitis. The exposure of MAC-T cells to *E. coli* culture filtrate or LPS induced increases in IL-1α, TNF-α and CXCL8 mRNA transcripts (153). Interestingly, the effect of culture filtrate was inhibited by polymyxin B, which neutralizes the lipidA component of LPS, indicating that LPS played a major stimulating role. Accordingly, the response of MECs to *E. coli* or LPS involves TLR4, and NF-κB is strongly activated (50). The signalling pathway implicates the TIR domain-containing adaptor myeloid differentiation factor 88 (MyD88), as shown by the inhibitory effect of a transdominant negative bovine MyD88 factor on pbMEC response to *E. coli* or LPS (50).

Numerous investigations of the response of MECs to bacterial stimuli have been done using gene microarrays or other high-throughput transcriptomic approaches, including more recently RNA-seq and extending to microRNAs (26, 136, 137, 139, 154). These approaches allowed investigators to compile lists of differentially expressed genes, and to delineate the putative signalling pathways behind the gene networks. Although the variety of protocols and in particular the use of different modes of stimulation (live or killed bacteria, culture filtrates or purified MAMPs) may account for different results, a few main features emerge from these studies. The major signaling pathways activated by exposure of pbMECs to killed *E. coli* or culture filtrate appear to depend on NF-κB, Fas, and AP-1 through IL-1α. Prominent among the MEC responses were the marked overexpression of multiple chemokines (CCL5, CCL20, CXCL8, CCL2, CXCL2, CXCL3, CXCL5, CX3CL1), illustrating a role of sentinel, and of antimicrobial molecules (BNBD, LAP, NOS2A, SAA3, CFB, HP), illustrating the role of defence effector fulfilled by MECs. Overexpression of chemokines generally precedes that of antimicrobial transcripts. This temporality has been related to different actions of the NF-κB and the CAAT box enhancer binding protein C/EBPβ transcription factors on the promoters of CXCL8 and LAP (155). A strong response of pbMECs to *E. coli* or LPS is the activation of the interferon signaling pathway (including of OAS1, MX1, MX2, ISG15, ISG20, IRF9, IFI44), and antigen presentation (MHC complex, CD74, proteasome PSMB9, PSMB8 and transporter TAP1) (26, 136, 137). This likely results from activation of the TRIF pathway downstream of TLR4 involvement. In addition, the transcription factor nuclear factor erythroid-2-related factor 2 (Nrf2), known for its role in cytoprotection to oxidative stress, has been shown to be activated in pbMECs exposed to killed *E. coli*, with transcriptional activation of 16 target genes (156). Experiments with siRNA-mediated knockdown of Nrf2 indicated that Nrf2 positively regulates the innate immune response of pbMECs to LPS. An important consequence of the activation of NF-κB and the production of TNF-α or IL-1β is the reduction of milk component synthesis, caseins, milk fat, and lactose (104, 150, 151).

Killed *E. coli* elicit an early differential regulation of miRNAs, some of them related to immunity (154). Several exosomal miRNAs are differentially expressed by MAC-T cells stimulated with *E. coli* LPS (157). One of them, miR-193b-5p, which was overexpressed, participates in the regulation of the NF-κB pathway, causing an increase in IL-6 mRNA and decrease in IL-1β, TNF-α, and TGF-β mRNA, indicating that this miRNA could be a regulator of LPS-induced inflammation (157). At the mammary tissue level, mir-223, known as a dampener of inflammasome formation, was induced early during *E. coli* infection, along with NF-κB inhibitors and the suppressors of cytokine signalling SOCS1 and SOCS3 (158).

It has been reported that the MG of cows infused repeatedly with *E. coli* endotoxin became partially resistant to systemic and local effects related to inflammation and reduced milk secretion, thus showing endotoxin tolerance (159). Although monocytes/macrophages are known to play a major role in endotoxin tolerance, other cells can contribute (160). Endotoxin tolerance usually tunes down the inflammatory response, while maintaining the antimicrobial response of the refractory cells. This stems from a comprehensive gene reprogramming that modulates the cell response to a second exposure to endotoxin, which can last up to 5 days (160). It has been shown that pbMECs are subject to endotoxin tolerance: a first exposure to *E. coli* endotoxin (LPS priming) tends to enhance the expression of bactericidal and immune-protective factors (such as the defensins BNDB4 and LAP, or MUC1) and to repress the expression of certain cytokines and chemokines (such as TNF-α, IL-6 and CXCL8) (161). Accordingly, bMECs could contribute to the control of the inflammatory response to *E. coli*, while participating in the local defence early during infection.

#### 4.1.2 Reacting to *S. aureus*

Most if not all studies concurred to find that the response of bMECs to *S. aureus* is of much lower magnitude and breadth compared to that to *E. coli* (121, 136, 137, 156, 162). This results in a much lower expression of mRNA for cytokines (TNF-α, IL-1β, IL-6), chemokines (CXCL8, CCL2, CCL5, CCL20), and antimicrobial molecules (NOS2, DEFB4/LAP, S100A9, LTF, CFB, C2, C3). This is also true of the response of bMECs to *S. aureus* LTA when compared to LPS (163). Exposure of pbMECs to *S. aureus* culture filtrate elicits a response from bMECs, even though moderate compared to the response to *E. coli* LPS (137). Secreted or released compounds such as hemolysin-alpha, staphylococcal protein A and LTA contributed to the stimulation of pbMECs.

The poor activation of MECs has been attributed to the subversion of the activation pathways. MAC-T cells exposed to *S. aureus* LTA are activated through the TLR2/MyD88/phosphoinositide 3-kinase (Pi3K)/AKT pathway (164). In this study, miRNAs in MAC-T cell exosomes were affected by the LTA stimulation. The miRNA miR-23a was shown to downregulate the inflammatory response by targeting PI3K. Importantly, it has been put forward that a major difference from activation by *E. coli* is that NF-κB is not induced by *S. aureus* (50). However, it was shown that NF-κB was not activated by killed *S. aureus* in the presence of FBS, but that activation occurred in the absence of FBS in the stimulation medium (94). MAC-T cells do not react to LTA or peptidoglycan fragments used alone, but co-treatment with both MAMPs differentially regulated various inflammation-related genes with the involvement of NF-κB (103). In a study showing that the response of pbMECs to *S. aureus* is dominated by the IL-6/IFN-β pathway, it was suggested that *S. aureus* subverted the Myd88-dependent activation of immune gene expression (136). This finding was extended to the mammary tissue (teat sinus), showing that the early response to *S. aureus* did not involve TLR signalling and NF-κB activation (158). This result would suggest that LTA and other staphylococcal components are not released in amounts sufficient to trigger a response from MECs during the early phase of infection. Another finding was that contrary to *E. coli*, *S. aureus* impairs Nrf2 activation, as none of the Nrf2 downstream genes activated by *E coli* were activated by *S. aureus* (156).

Contrary to most *E. coli* strains, *S. aureus* can invade MECs, as shown *in vitro* using MAC-T cells, the cell line of choice to study invasion mechanisms, as pbMECs are less permissive (165, 166). *S. aureus* modulates the actin-cytoskeleton through activation of RhoA GTPases, which correlates with the capacity of *S. aureus*, but not *E. coli*, to invade MAC-T cells (158). There was no indication that this invasion induces inflammatory signals from the infected cells (134). Consequently, the outcome could be either elimination of the infected MEC by sloughing and shedding at milking, inactivation by lysosomal antimicrobial compounds, or shielding from professional phagocytes or antimicrobials, thus favouring chronic infection. Another possibility could be apoptosis followed by efferocytosis by ductal macrophages, with or without killing of bacteria. This issue remains an open question. In a recent study it has been shown that the invasion of MAC-T cells by *S. aureus* induces activation of the NLRP3 inflammasome and caspase-1 (67). This is followed by the pyroptosis (inflammatory necrosis) of the cells and release of mature IL-1β and IL-18, thus enhancing inflammation.

The responses of MECs to *S. aureus* have been reported to vary among strains in terms of magnitude (32) or transcriptomic signatures (167). Different responses to culture filtrates from different strains were also reported (137). Differences were noted as a function of bacterial components or clonal complex types (168), or surface expression of adhesion proteins (169). However, it has been reported that stimulation in the presence of 10% FCS suppressed strain differences (34). It is also important to bear in mind that culture filtrate or heat-killed *S. aureus* produce biological effects that are essentially different from those induced by live bacteria (42). The comparison of the results obtained with different experimental protocols is not straightforward. It appears that dead bacteria are poorly recognized by MECs, and induce dampened immune responses. Live bacteria are likely to be more actively recognized and stimulating, but this could be essentially through the release of bacterial components, which needs prolonged co-culture of MECs with bacteria and exposes to experimental complications relating to bacterial overgrowth.

#### 4.1.3 Reacting to *S. uberis*

Interactions of *S. uberis* and MECs during infection is facilitated by adhesion of bacteria to MECs followed by internalization, as has been shown to occur *in vitro* (14, 170). Experimental infection of the bovine MG with *S. uberis* elicited differential expression of more than 2200 genes compared with control uninfected quarters (97). Among these genes, upregulation of those related to the immune response (IL-1β, IL-6, TNF-α, TLR2, CXCL8, SAA3, lactoferrin, complement C3) and downregulation of the major milk protein genes were prominent. In contrast, bovine mammary epithelial cells in culture challenged with the *S. uberis* strain used to induce clinical mastitis in the *in vivo* experiment did not cause a change in the mRNA levels of the immune-related genes. The contribution of MECs to the immune response during MG infection by *S. uberis* was established by monitoring the time-course of infection of the MG of ewes using immunohistochemistry of mammary tissue and proteomics analysis of milk fat globules (MFG), which are shed from MECs (171). Upon infection, MFG proteins were strongly enriched in antibacterial components such as lactoferrin, calprotectin and cathelicidins, and depleted in caseins. The MEC origin of these immune defence molecules was confirmed by immunohistochemistry analysis. Similar results were obtained upon experimental mammary infection of cows with *S. uberis*, confirming the involvement of MECs in the innate immune response to this pathogen (172). From the above experiments, it appears that MECs respond to *S. uberis* during MG infection, but the activation pathways, either by direct interaction with *S. uberis*, or as a consequence of other epithelial cell inflammatory responses, has not been established.

A thorough study of the interactions of *S. uberis* with pbMECs has established that these bacteria do not induce a noticeable immune response, whether alive or killed, encapsulated or not (41). Omitting serum from the stimulation medium did not make a difference. Live *S. uberis* induced some response on the part of bpMECs, but low compared to that induced by *E. coli*. *S. uberis* strains, live or killed, do not activate substantial TLR2 and NF-κB signalling in MECs and in HEK 293 cells transfected with bovine TLR2. Purified LTA from *S. uberis* significantly induced a response from boMECs, depending on NF-κB, thus suggesting the involvement of a PRR. However, as *S. uberis* LTA did not activate HEK293-TLR2, TLR2 would not be the PRR involved. Purified lipoproteins and glycolipids from *S. uberis* did not activate bpMECs. The authors concluded that LTA is not recognized on intact *S. uberis* bodies, likely because it is masked by other bacterial components or presented in a way that does not permit interaction with PRRs. Interestingly, *S. uberis* was able to stimulate bovine monocyte-derived macrophages and the murine macrophages of cell line RAW 264.7, as shown by the activation of NF-κB and the overexpression of TNFα, IL-6, Cxcl2 and Ccl5 mRNA as well as did dead *E. coli*. It seems that macrophages are able to “unpack” the MAMPs of *S. uberis* so that the PRRs are stimulated, and NF-κB activated.

The reported absence of reactivity of bMECs to *S. uberis* is at variance with other studies that found activation through commonly activated pathways. When a mouse mammary cell line (EpH_4_-Ev) was exposed for 3 h to live bacteria (of the widely used strain of *S. uberis* 0140J) with a multiplicity of infection (MOI) of 10, a number of cytokines were found (protein array) in the culture supernatant (TNF-α, IL-1β, IL-6, G-CSF, IL-2, IL-15, MCP-1) and the NF-κB DNA binding activity (electrophoretic mobility shift assay) was enhanced (173). The authors used the same cell line and *S. uberis* strain to establish that both TLR2 and TLR4 were involved in the detection of the bacteria, TLR2 being the principal detector, and that both the TLR/NF-κB and phosphatidylinositol 3-kinase PI3K/Akt/mTOR activating pathways were involved (174). These findings may be peculiar to the cell line used. The same group used bovine MAC-T cells in an in-depth study of activation pathways (175). They found that *S. uberis* induced an intracellular Ca^2+^ release in MAC-T cells, with activation of the PKCα/NF-κB and nuclear factor in activated T cells (NFAT) signaling pathways. Notably, translocation of NF-κB into the nucleus was detected by confocal microscopy. Secretion of the cytokines TNF-α, IL-1β and IL-6 was induced by *S.uberis*, along with cytoplasmic reactive oxygen species (ROS). The authors concluded that *S. uberis* induced inflammatory responses through lipid products mainly by IP3 (inositol triphosphate) that acts on membrane phosphoinositides and activates the NF-κB and NFAT pathways.

There is no consensus on the direct activation of MECs by *S. uberis*, as the divergent results obtained by the two research groups that dealt with the issue are difficult to reconcile. It should be noted that, regardless of the mastitis-associated pathogen, most data come from *in vitro* studies of the response of MECs to bacterial stimuli, so interactions that occur *in vivo* with other cell types are not satisfactorily taken into account.

### 4.2 Cooperative reaction with macrophages and intra-epithelial lymphoid cells

**Figure 4:**
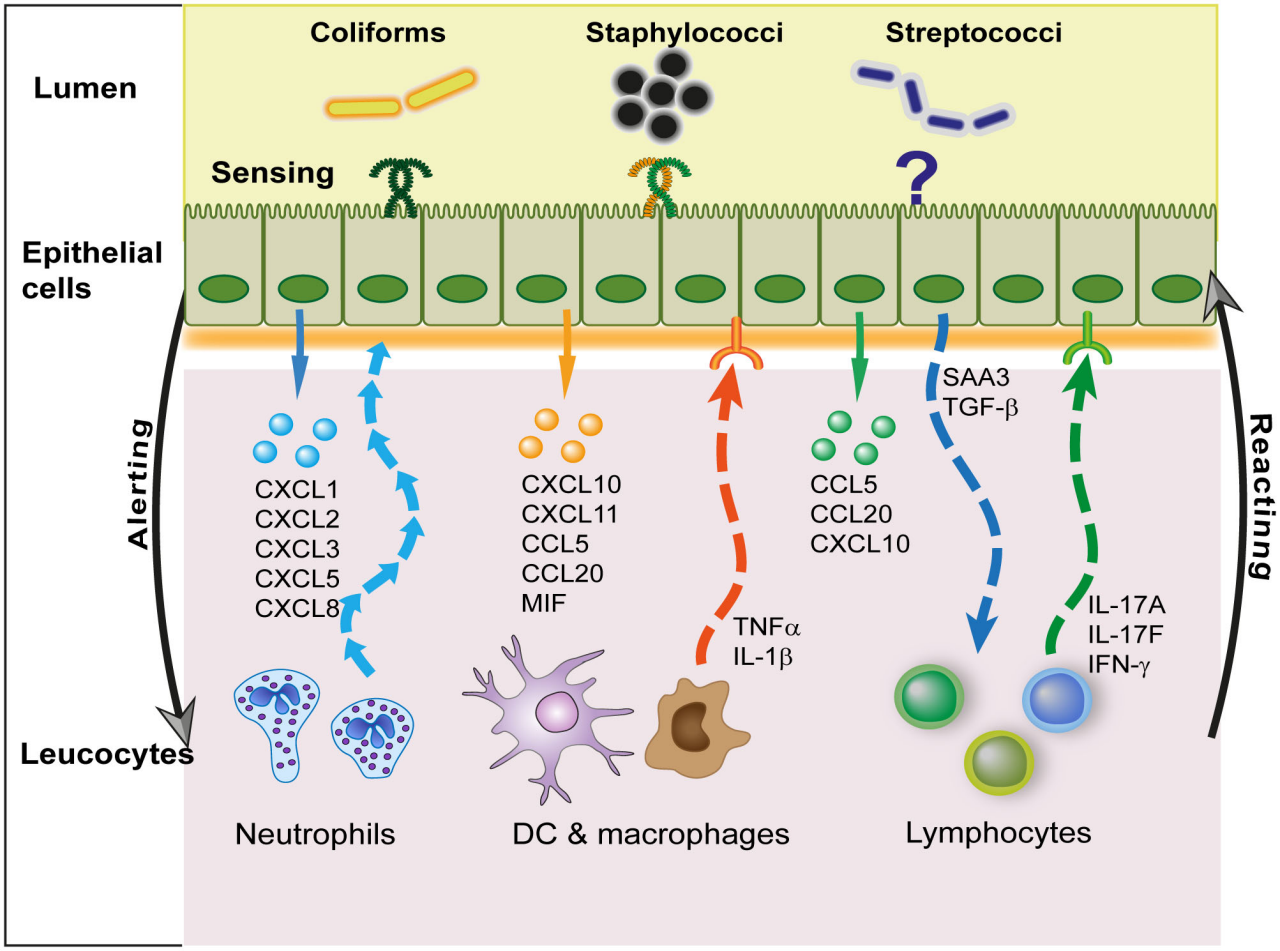
Interactions of MECs with leucocytes. The main communication activity of MECs with leucocytes is mediated by the secretion of chemokines that target preferentially either neutrophils, mononuclear cells (dendritic cells and macrophages), or lymphocytes. In this way, they fulfill an important alerting role. In turn, leucocytes release pro-inflammatory (TNF-α, IL-1β) or modulatory (IL-17, IFN-γ) cytokines that modify the response of MECs to infection. Epithelial cells may also modulate leucocyte activity *via* the release of local molecular cues such as serum amyloid A3 (SAA3) or TGF-β.

It is likely that macrophages have bi-directional interactions with epithelial cells (**Figure 4**). The response of bMECs to exposure to bacteria does not exactly recapitulate the early response of the MG to infection (26, 97). The recognition of *S. uberis* by macrophages but not by pbMECs has already been mentioned (41). The response of bMECs to *S. uberis* during infection strongly suggests that leucocytes stimulate MECs (172). The production of inflammatory cytokines by MECs is not well documented at the protein level, as most studies reported only the overexpression of cytokine transcripts. Examples of induced TNF-α and IL-1β transcription without protein secretion by bMECs have been reported (33, 176). The low production of TNF-α by MECs *in vitro* contrasts with the early increases in TNF-α in *E. coli* mastitis (32, 177). Epithelial (ductal) macrophages may be the main source of pro-inflammatory cytokines in the immediate and early phase of MG inflammation. The increase in mammary tissue expression of the *IL10* gene in response to *E. coli* revealed the contribution of leucocytes, possibly macrophages, resident or recruited at 3 h post-challenge (29, 178). Bovine mammary macrophages could contribute to the virulence of *S. uberis* for the MG (179). Oxylipids are potent lipid mediators of inflammation, which are produced in the mammary tissue during infection (180, 181). They could participate in the MEC-macrophage communication. Exosomes released by MECs are another possible communication vehicle with macrophages, through the miRNA they contain (101).

All these observations suggest a close cooperation between intra-epithelial (ductal) macrophages and MECs, consistent with their close physical association (8, 10). This underexplored area of research merits further investigation. Another field of research is the role of intra-epithelial lymphocytes, mainly CD8^pos^ cells that populate the mammary epithelial lining. Their constant presence and memory cell phenotype have a biological significance yet to be discovered.

## 5 Epithelium interactions with lymphocytes

Although there is no organised lymphoid formation in the healthy MG, except in the Furstenberg’s rosette of infected glands, a few lymphocytes are scattered within the epithelium and in the connective sub-epithelial tissue (6, 182, 183). In the lactating mouse MG, only 2% of leucocytes (CD45^pos^) are B lymphocytes and 10% T lymphocytes (10). During infection, the mammary tissue is infiltrated by large numbers of recruited lymphocytes shortly after or concomitantly with the initial influx of neutrophils. Lymphocytes can be seen between epithelial cells, sometimes associated with damaged epithelial cells or even within swelled cells, suggesting that they actually had caused the damage (147). A few publications report the influx of lymphocytes into milk shortly after the onset of inflammation (183). Activated T lymphocytes are constantly excreted in milk from healthy glands (184), and T lymphocytes constitute a sizeable proportion of milk leucocytes of MGs infected with streptococci or staphylococci (185, 186). Milk lymphocyte counts began to increase as soon as neutrophil counts increased after experimental infection of the MG with *E. coli* (187). The recruitment of CD8+ lymphocytes was concomitant with an increase in somatic cell count in milk (mainly neutrophils) after infusion of staphylococcal α-toxin into the lumen of the MG of sensitized cows (188).As the recruited lymphocytes have necessarily crossed the epithelium, they must have interacted with mammary epithelial cells.

When properly stimulated, MECs produce chemokines that attract mononuclear leucocytes. In particular they secrete CCL20 at the onset of mastitis (140, 178), a chemokine that targets the chemokine receptor CCR6, expressed notably by lymphocytes poised to migrate to sites of epithelial inflammation (189). CCL20 also attracts naïve and mature DCs, which enables it to play a role in adaptive immunity.

These observations lend support to the crosstalk between MECs and lymphocytes through both soluble mediators and direct contact. In other epithelia such as the intestine, intraepithelial lymphocytes (IELs) are sentinels of the mucosal barrier (190). The exchanges between MECs and leucocytes are bidirectional (**Figure 4**). Upon detection of bacteria, MECs secrete chemokines that attract leucocytes, and in turn leucocytes secrete interleukins (monokines, lymphokines) that modulate MECs responses to bacteria. MECs are equipped to communicate with lymphoid cells. MECs express the receptors and are able to respond to lymphokines, cytokines produced by lymphocytes, such as IFN-γ and IL-17A or IL-17F (28, 176, 191, 192). They can also react to inflammatory cytokines such as IL-6 and TNF-α (193–195), the latter in synergy with IL-17 (176). These cytokines modulate the response of MECs to micro-environmental cues such as MAMPs. This has been documented for *S. aureus* LTA or MDP, showing with IL-17A and IL-17F a synergistic increase in the expression of the cytokines CXCL8, CCL2 and CCL20 or the AMPs S100A8 and TAP (176). The response of MECs to *E. coli* or LPS is also augmented by IL-17A (28). Epithelial tight junctions are regulated by cytokines such as TNF-α and IFN-γ (196). The lymphokines IL-17 and IL-22 are known to regulate intestinal epithelial permeability (197), an activity that remains to be established in the MG. In the lung, resident memory CD4^pos^ T cells producing IL-17 fine-tune epithelial functions, hastening the innate immune response including neutrophil recruitment during pneumonia (198). Epithelial cells could also influence lymphocytes. Constitutive production of TGF-β by MECs and its increased production during mastitis is also likely to modulate leukocyte responses (199). Among modulating molecules highly produced by activated MECs, SAA3 may modulate the immune response to infection, as SAA is known to modulate Th17 lymphocytes (200). Overall, we know very little about the reciprocal interactions of lymphocytes and MECs during MG infection.

Besides its reaction to MAMPs, the MG can react to non-pyrogenic antigenic molecules. The intramammary infusion of ovalbumin into the lumen of uninflamed MGs elicits an intense neutrophilic inflammation only in cows previously sensitized to this antigen (201). This inflammatory response is associated with the local production of IL-17A and IFN-γ and depends on the induction of CD4+ Th17 cells by immunisation (202, 203). This mammary antigen-specific response (mASR) supposes that at steady state (without inflammation or epithelial leakiness to explain the paracellular passage of ovalbumin, a 40 kDa protein of 4 nm in diameter) the mammary epithelium is able to sample the lumen, take up an antigen, process it, and present it to resident memory lymphocytes that in turn trigger an inflammatory response on the part of the epithelium (183). The mASR can synergise with the innate immune response to MAMPs (77), so that it can be put forward that this type of immunity could amplify the response to infection and be a vaccine target (204). That the mammary epithelium not only senses MAMPs, but also antigens, raises the question of the presence of APCs in the MG epithelium. DC-like cells have been identified in the epithelium lining milk cisterns and ducts (7), and we know that sub- or intra-epithelial DCs can project dendrites between ECs, sharing tight-junction-like structures with them, and sampling the lumen content, as shown in the intestine (205). It is thus plausible that bacteria and bacterial antigens can be presented to resident memory T cells close to the epithelium. However, the existence of DCs or macrophage transepithelial dendrites is undocumented in the MG, and even in the intestine may be induced by the microbiota and luminal pathogens (206). The ductal macrophages are ideally located and might be mammary APCs. This remains to be documented, so it is important to note that the representation of ductal macrophages dendrites sampling the lumen of the mammary gland in **Figure 3** is speculative only.

The question of antigen-presentation by MECs can be posed, as epithelial cells are able under certain circumstances to present antigens to CD4+ or CD8+ lymphocytes (206). Intestinal ECs can take up luminal soluble proteins through pinocytosis, process them to antigenic peptides and present peptides in association with MHC class II at the basolateral face. This antigen trafficking is enhanced by IFN-γ (207). The bovine MG has a low constitutive expression of MHC class II expression, but class II molecules can be induced by intramammary infusion of killed bacteria (208). *In vitro*, bovine MECs can be induced to express MHC class II molecules by incubation with IFN-γ (209). When stimulated by MAMPs, bovine MECs overexpress co-stimulation molecules, which are necessary for efficient interaction with lymphocytes, such as CD83 (137). Thus, presentation of antigens to resident memory lymphocytes by MECs is not established but cannot be excluded in our present state of knowledge.

## 6 Intervention strategies targeting the MG epithelium

The effects of a plethora of products on the response of MECs to bacteria or bacterial products have been documented over the past two decades. Due to space constraint, we will only mention a few categories of interventions, illustrating them with a limited selection of publications. References can also be found in (210). A reminder seems useful: infection of the MG generally causes mastitis, that is to say MG inflammation. It is tempting to consider that mastitis control can be achieved by alleviating “aberrant” inflammation (211). Some studies have shown in mouse models of LPS or *E. coli* mastitis that a reduced TLR4-dependent inflammation alleviates the severity of mastitis (discussed in [212)]. The implicit rationale is that as mastitis is by definition an inflammation of the MG, abating inflammation will suppress mastitis. Nevertheless, even though inflammation in excess exerts collateral tissue damages, it is also necessary to control the exuberant proliferation of bacteria that will make mastitis a potentially fatal disease (213, 214). Ideally, the inflammatory reaction should be prompt, intense at the onset, effective, short-lived, followed by a rapid resolution phase. With some pathogens, the effectiveness is not achieved, this causes clinical and chronic mastitis. It should be kept in mind that the MG can afford a sustained degree of inflammation, owing to the quenching effect of milk on reactive oxygen species and proteases, and its high capacity of regeneration over the lactation cycle (215). It may be possible to fine-tune the MG reaction threshold, but so far implementation and real-life evaluation of the numerous *in vitro* or small-scale studies that tested means to reduce or enhance the alertness and reactivity of the MG to infection are awaited.

### 6.1 Corticoids and non-steroid anti-inflammatory drugs

The use of corticosteroids to treat mastitis has long been considered a double-edged sword, limiting inflammatory tissue damage on one side, but dampening the effectiveness of immune defences on the other. Intramammary administration of prednisolone during mastitis protects the blood-milk barrier, decreasing the recruitment of blood components such as immunoglobulins and cells, potentially decreasing the local defences against infection (216). The manipulation of the mammary blood-milk barrier is discussed in (144). Intramammary administration of the nonsteroidal anti-inflammatory drug (NSAID) ketoprofen reduces the response of the MG and MECs *in vitro* to LPS (217). Meloxicam, an inhibitor of cyclooxygenase-2 an enzyme that generates prostaglandins, also reduces the inflammatory response of MECs to *E. coli* LPS and staphylococcal LTA (113). Different NSAIDS have somewhat different effects on the MEC barrier recovery after a challenge with LPS (218). The net effect of these drugs on immune defence in the MG, elimination of the pathogen and recovery of secretory function, remains to be further investigated.

### 6.2 Active principles of medicinal plants and dietary components

Testing the effect of herbal extracts on MECs *in vitro* is a popular topic, which is the subject of numerous publications. For example, curcumin attenuates LPS inflammation in a mouse model of mastitis (219), but represses casein synthesis by MECs (220). Avocado leaf lipid extract (221), lotus leaf (222), *Thymus vulgaris* (223), Tea tree oil (224), Dandelion (225), Stevia (226), Orange oil (227), vanillin (228), *Panax ginseng* (229), and many others could be mentioned. Vitamin D reduced invasion of MECs by *S. aureus*, interfering or not with the cell defence response yue (230, 231). Sodium octanoate (a medium chain fatty acid) interferes with the internalization of *S. aureus* by MECs by modifying the expression of α5β1 integrin, TLR2, and CD36 (232).

These studies and many others present promising effects and many improve our knowledge of the intracellular response mechanisms of MECs (230, 231). However, their main practical limitation is that the safety of the various extracts and their long term effect on the MG are not investigated *in vivo*. Their use has therefore not yet passed into common practice.

### 6.3 Intramammary probiotics

Probiotic bacteria, which are mainly lactic acid bacteria (LABs), share with commensals and pathogens several MAMPs. They can interact with the PRRs of the mammary epithelium, and indeed, they induce inflammation when introduced into the MG (233). This precludes their extensive use as a preventative tool for MG infections, as it would result in high bulk tank milk SCC. However, the propensity to induce inflammation and stimulate the local innate immune response could be exploited to cure MG infections. Intramammary administration of a probiotic strain of *Lactococcus lactis* proved to be as effective as a common antibiotic formulation to cure naturally acquired MG infections (234, 235). The authors suggested that the cure resulted from the induced local inflammation, including an intense recruitment of leucocytes, and stimulation of MG defences. Interestingly, the lactococci were eliminated from the treated gland in a few days. If confirmed, this approach would be an alternative to antimicrobials in order to treat clinical and subclinical mastitis. The effects of suitable LABs on the immune stimulation of the mammary epithelium and the impact on milk secretion deserve further studies.

### 6.4 Non-specific immunomodulation

It took experimenters some time to realize that to experimentally elicit an IMI with few bacteria and high probability, it was necessary to select MGs that shed low numbers of leucocytes, because high SCCs prevent infection [discussed in (100)]. We now know that besides milk leucocytes, the stimulation of MECs contributes to the refractory property of inflamed MGs. The overexpression of PRRs could lower the threshold of alertness of the mammary epithelium, shortening the lag phase that precedes reaction to bacterial intrusion into the MG lumen. The instillation of recombinant bovine sCD14 into the MG increased the response to intramammary LPS and reduced the severity of *E. coli* infection of lactating cows (236). This increase in the alertness or reactivity of the mammary epithelium was accompanied by a more rapid recruitment of leucocytes into the MG lumen. Although not documented, it is possible that overexpression of PRRs by MECs allow these cells to better resist bacterial invasion, since the alteration of signalling pathways associated with PRRs makes mouse MECs prone to invasion by bacteria (*E. coli* mastitis-associated strains) that normally do not manifest invasive abilities (237, 238). Another possibility is the priming of the epithelium, as described following small amounts of LPS, that is characterized by a contained inflammatory response but a heightened self-defence response to a consecutive challenge with *E. coli in vivo* (239) or *in vitro* (161). LPS is not the only MAMP that can modify the immune response of MECs. Bovine MECs can also be primed with Pam2CSK4 (TLR2/6 agonist) (240). Pre-treatment of rat MECs with β-glucan (an agonist of Dectin-1) dampened the inflammatory response of these cells to LPS (65). The reprogramming of MECs is only transient and wanes in a few days (241). It has been shown that the responsiveness of the leucocytes (macrophages and lymphocytes) associated with the lung epithelium can be maintained long after the initial infection is cleared (242). This has not been established in the MG, and may even be questioned if one considers that MG infections generally elicit imperfect protection against recurrence [discussed in (243)]. The pre-conditioning with LPS or LTA of the mouse MG to *S. aureus* infection would not involve alveolar and epithelial macrophages, as their inactivation with clodronate does not prevent immunomodulation (244). Pharmacological activation of epithelial TLRs may confer significant protection, as has been shown in the context of lung resistance to bacterial infections (245).

### 6.5 Vaccination

It is in principle possible to harness vaccination with a view to protecting the MG epithelium against bacterial colonisation. Adherence to and invasion of MECs is a strategy for bacterial colonisation of the MG, implicated in the persistence of infection. Preventing adherence with antibodies to bacterial surface adhesins could thus be an effective counter-measure. An example is the induction of antibodies to the surface *S. uberis* adhesion molecule (SUAM). Antibodies obtained by immunizing cows with recombinant SUAM reduce adherence to and internalization of *S. uberis* by MAC-T cells (246). However, protection against experimental infection was limited (247), suggesting that other antigens would be needed for an effective vaccine. A more general limitation of this approach to combat mastitis is the dilution of antibodies by milk during lactation, and the limited capacity of the bovine MG to produce locally antibodies (248).

Another mechanism of epithelium protection through vaccination is the effect of lymphokines on the autonomous defences of MECs. In conjunction with MAMPs and inflammatory cytokines (TNF-α, IFN-γ), Th17 cytokines stimulate the expression of immune defence genes (28, 176). In response to these cytokines, both epithelial self-defence (antimicrobial peptides and enzymes) and leukocyte help (recruitment by chemokines) are enhanced. Moreover, vaccination could seed the mammary epithelium and sub-epithelial stroma with resident memory lymphocytes, with the potential of a quicker and more effective immune response than before immunization (183). It is therefore tempting to view vaccination as a tool to reprogram the response of the mammary epithelium to infection by mastitis-associated bacteria.

## 7 Knowledge gaps and prospects

Knowledge of the mammary epithelium activation pathways in response to bacterial stimuli is accumulating, but a comprehensive view is difficult to conceive. This partly results from the uneven coverage of the research domain, which leaves sections in knowledge deficit despite their theoretical importance. The ease of *in vitro* culture of MECs has led to intensive use of this study model and to highlighting the role of epithelial cells in an isolated situation. More emphasis could now be placed on the interactions of polarized MECs with leucocytes. Interactions of ductal macrophages with MECs deserve particular attention. Resident macrophages, through the release of TNF-α and IL-1β, could stimulate NF-κB-dependent MECs response, even when ECs do not respond directly to the bacteria, as it has been shown in the lung in the setting of pneumococcal infection (249). The study of ductal macrophages in relation to the triggering of the inflammatory reaction, its modulation and its resolution, then tissue repair, will bring great advances in the understanding of the innate immune defences of the udder. The same consideration applies to the role of resident memory CD8 intraepithelial T cells. To study cellular interactions, a more intensive use of mammary tissue explants should be done to complement the *in vitro* MEC models. Along with the immunohistochemistry approach, the roles of the epithelium would be studied with more fidelity and depth than with the simple MEC model grown on a solid or porous surface. These techniques would help fill some of the many knowledge gaps that remain despite an already considerable research corpus.

On the side of the stimuli to the mammary epithelium, the concept of vita-PAMPs, molecules produced by viable but not dead microbes, pathogens or not, such as mRNA or quorum sensing molecules (110, 250) is to be considered. Detection of microbial viability by the mammary epithelium deserves to be investigated. What is to be detected and needs a prompt response is metabolically active bacteria, since bacterial growth can be rapidly overwhelming in lactating MGs. We know little of the PRRs that contribute to the response of the epithelium to vita-PAMPs (251).

Modulation by lymphocytes of epithelium response to infection and management of the inflammatory response followed by resolution and tissue repair deserves more interest. Our knowledge of the effects of cytokines, and particularly the lymphokines IFN-γ, IL-17A, IL-17F, IL-22, and IL-26, on the epithelium immune defences needs improvement. These lymphokines can be produced by resident or recruited memory lymphocytes elicited by infection or vaccination and could be harnessed to control mastitis. Cell-cell contact dependent interactions between MECs and intraepithelial leucocytes (macrophages, CD8^pos^ T cells) are also of interest. The roles of innate lymphoid cells and mucosal-associated invariant T (MAIT) cells, recently identified in human and bovine milk (252, 253), merit investigation.

**Figure 5:**
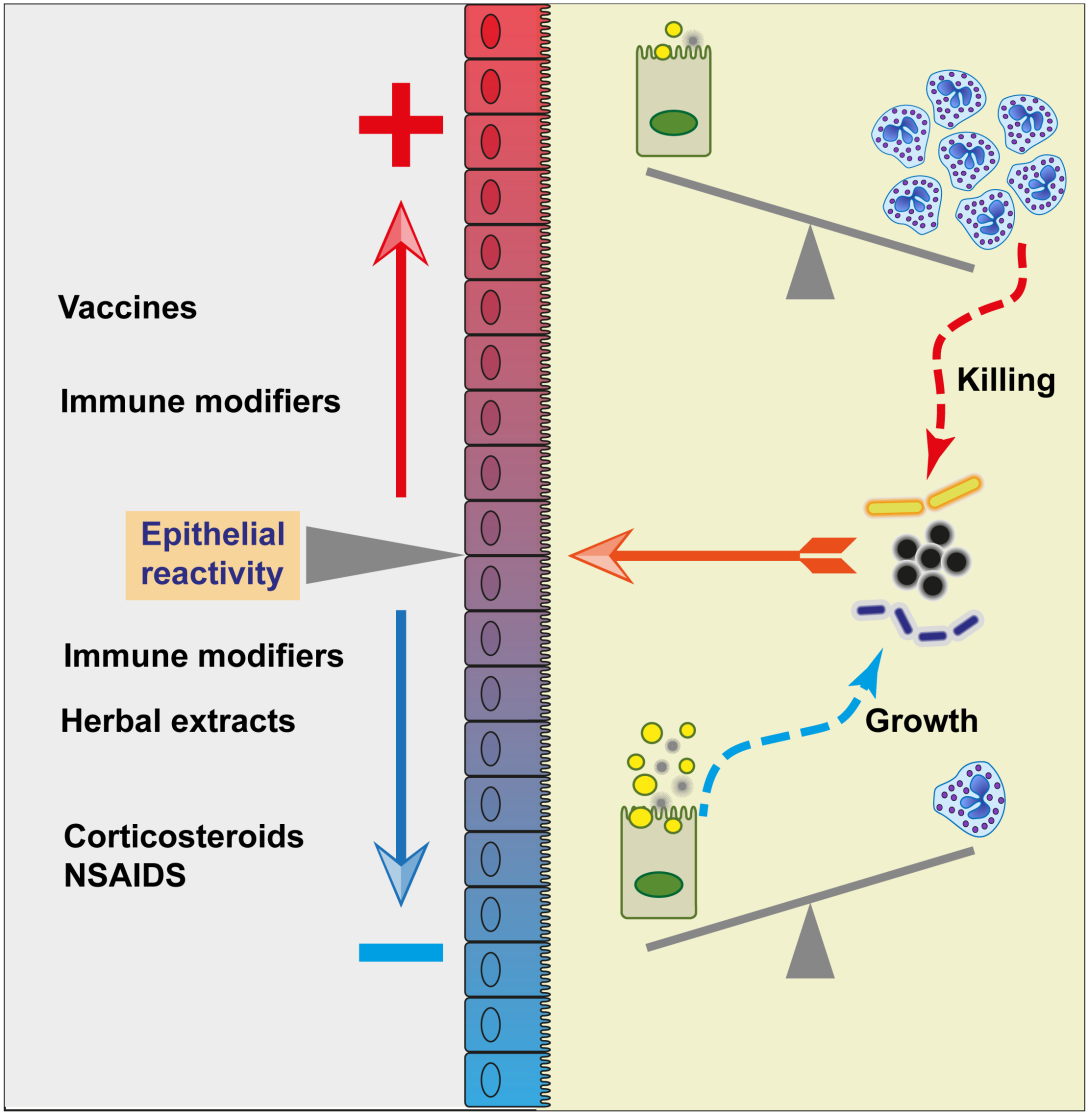
Manipulating the reactivity of the mammary epithelium. Upon contact with bacteria and bacterial products (MAMPs, metabolites), the mammary epithelium reacts according to the intensity of the stimulus and its degree of alertness (reactivity). A high reactivity will lead to a strong inflammatory reaction characterized by a large influx of neutrophils, to the detriment of the secretory function of MECs but with the advantage of killing the bacteria. A weak reactivity will lead to a reaction of less intensity, recruiting fewer neutrophils and sparing the secretion of milk components (represented here by fat globules and casein micelles). As a result, bacteria can proliferate in milk. In principle, it is possible to manipulate the alertness and reactivity of the mammary epithelium. Nonspecific immunomodulation with immune modifiers could either increase or decrease the epithelial reactivity, whereas vaccination tends to increase the specific immune response to bacteria. Anti-inflammatory drugs (corticosteroid or NSAIDS) would reduce the inflammatory response, an effect also sought with herbal extracts.

A major goal of mammary immunity research is to improve the resistance of the udder to infection. One way to modulate MG defences is to adjust its “reaction threshold” to the local microbial stimulus (**Figure 5**). When the magnitude of stimulus crosses a certain threshold, defence reactions are triggered. In principle, graded responses match up with stimulus intensity. What determines the threshold is the degree of alertness of the epithelium on one hand, and the properties of the pathogen on the other hand. The mammary epithelium must be responsive to microbial stimulation because of the threat posed by the proliferation in its lumen of bacteria that thrive in a nutritious growth medium, which exposes lactating mammals to potentially fatal infections. We can infer that the default setting of the reaction threshold to MAMPs is tuned to render the mammary epithelium rather reactive. However, overreacting would endanger the secretory function of the MG. Consequently, a graded response of the MG epithelium to infection is appropriate. It appears that “ordinary” MAMPs are sufficient to elicit a first level inflammatory response which favours self-defence of the epithelium and production of chemokines, but with low amounts of inflammatory cytokines (TNF-α, IL-1β). The first inflammation level is sufficient to recruit neutrophils, with moderate activation and plasma exudation, which means little tissue damages but also moderate killing efficacy. When vita-PAMPs or aggressive virulence factors (pore-forming toxins, bacterial injection systems, cellular invasion) are detected, a full-blown inflammation is triggered, involving inflammatory cytokine production and inflammasome activation, with enhanced antimicrobial efficacy at the price of more tissue damage. According to the circumstances, it could be useful to lower the threshold of alertness of the gland or, conversely, to increase this threshold in order to remain at the first level of inflammation. Manipulating the reactivity of the mammary epithelium is a promising way of dealing with the mastitis issue, but more research is needed to put it into practice.

## 8 Conclusions

The MG is a peculiar immunological niche, endowed with the task of providing passive immune protection to the young, nurturing it for a long period, while preventing the overgrowth of bacteria in its lumen. In this review, we examined how the mammary epithelium manages to meet the latter requirement. We have not addressed the broader context of physiological stages (gestation, parturition, lactation) with their hormonal influences, and nutrition (e.g. effects of negative energy balance (122) [254)] on the reactivity of the mammary epithelium. However, these influences are far from negligible and would deserve a more comprehensive review. Regarding the interactions of the mammary epithelium with mastitis-associated bacteria, and despite a few discrepancies in the published results, divergences in interpretations, and remaining blind spots, some trends can be discerned:

- The mammary epithelium is not isolated from the lumen content by a gel-like mucus layer. Thus, the epithelium is directly exposed to bacteria and their exported constituents (membrane vesicles, secreted toxins, enzymes, metabolites and MAMPs).
- Dilution in large volumes of milk followed by removal at milking make the common armamentarium of mucosal surfaces ineffective in the MG: during lactation, secretory IgA or other antibodies and antimicrobial peptides remain below biologically active concentrations.
- The first line of defence against intramammary bacteria is the epithelium lining made up of MECs and ductal macrophages. MECs have been established as sentinels of the MG. The contribution of ductal macrophages remains to be documented.
- MECs, through their capacity to respond to lymphokines such as IFN-γ and IL-17A or IL-17F, they are effector cells of adaptive immunity.
- MECs operate in a complex network of interactions with local and recruited leucocytes during infection. This is this network that needs to be explored if we intend to devise tools to improve effectively MG defences and control the mastitis issue.
- A promising way to modulate MG defences is to adjust the “reaction threshold” to the local microbial stimulus.

## Author contributions

PR: Conceptualization, writing, original draft preparation. FG: Conceptualization, review and editing. PG: Conceptualization, review and editing. All authors contributed to the article and approved the submitted version.

## Funding

This work was supported by Institut National de Recherche pour l’Agriculture, l’Alimentation et l’Environment (INRAE) and the Eger program of APIS-GENE (Masticells project).

## Conflict of interest

The authors declare that the research was conducted in the absence of any commercial or financial relationships that could be construed as a potential conflict of interest.

## Publisher’s note

All claims expressed in this article are solely those of the authors and do not necessarily represent those of their affiliated organizations, or those of the publisher, the editors and the reviewers. Any product that may be evaluated in this article, or claim that may be made by its manufacturer, is not guaranteed or endorsed by the publisher.
